# The Association of Hypertension With Posterior Reversible Encephalopathy Syndrome in Systemic Lupus Erythematosus Patients: A Systematic Review

**DOI:** 10.7759/cureus.50620

**Published:** 2023-12-16

**Authors:** Japneet K Bhangu, Khalid Javed, Prabhleen Kaur Manshahia, Shamsun Nahar, Srishti Kanda, Uzair Chatha, Victor A Odoma, Aakanksha Pitliya, Esraa M AlEdani, Safeera Khan

**Affiliations:** 1 Internal Medicine, California Institute of Behavioral Neurosciences & Psychology, Fairfield, USA; 2 Anesthesiology, California Institute of Behavioral Neurosciences & Psychology, Fairfield, USA; 3 Internal Medicine/Family Medicine, California Institute of Behavioral Neurosciences & Psychology, Fairfield, USA; 4 Medicine, All India Institute of Medical Sciences, Rishikesh, IND; 5 Internal Medicine, Jean-Charles Medical Institute, Orlando, USA; 6 Medicine, California Institute of Behavioral Neurosciences & Psychology, Fairfield, USA; 7 Research, California Institute of Behavioral Neurosciences & Psychology, Fairfield, USA; 8 Cardiovascular/Oncology (Acuity Adaptable Unit), Indiana University Health, Bloomington, USA; 9 Dermatology, California Institute of Behavioral Neurosciences & Psychology, Fairfield, USA

**Keywords:** headache, hypertension, posterior reversible encephalopathy syndrome, reversible posterior leukoencephalopathy syndrome (rpls), systemic lupus erythematosus

## Abstract

Posterior reversible encephalopathy syndrome (PRES), also known as reversible posterior leukoencephalopathy syndrome (RPLS), is a rare disorder that most commonly affects the posterior part of the brain. Two common causes of PRES are hypertension and autoimmune diseases such as systemic lupus erythematosus (SLE). This systematic review followed the Preferred Reporting Items for Systematic Reviews and Meta-Analyses (PRISMA) 2020 recommendations and aimed at finding the association between hypertension and PRES in SLE patients. We searched medical databases such as PubMed, PubMed Central (PMC), Cochrane Library, and Multidisciplinary Digital Publishing Institute (MDPI) for relevant medical literature. The identified papers were screened, subjected to inclusion and exclusion criteria, and ran through quality appraisal tools, after which 16 papers were finalized. The finalized papers explored the roles of hypertension in SLE patients diagnosed with PRES. In this review, we identified a link between hypertension and PRES-SLE patients. We aimed to explain the role of hypertension in the development of PRES in SLE patients. This study also explains the different treatment modalities to be used for treating the patients presenting with PRES and differentiates other neuropsychiatric illnesses commonly present in SLE patients from PRES. It's important to make an accurate clinical diagnosis by understanding the clinical features and neuroimaging results of PRES for future care since it may even be incurable in some circumstances.

## Introduction and background

Reversible posterior leukoencephalopathy (RPLS) was first identified by Hinchey et al. in 1996 [1]. To emphasize the shared involvement of gray and white matter in RPLS, Casey et al. coined the name posterior reversible encephalopathy syndrome (PRES) in 2000 [2]. PRES is a neurologic condition that is rapidly developing and has distinctive clinical and radiological characteristics [3]. White matter edema that is reversible and primarily affects the back of the cerebral hemispheres is a hallmark of PRES [4]. Headache, seizures, visual problems, decreased mental function and nausea are typical manifestations with headache and seizures being the most frequent symptoms [5,6]. The major imaging finding during the acute stage is vasogenic edema in the subcortical parietal-occipital white matter [7]. It has also been reported that PRES affects other parts of the brain, including the brain stem, cerebellum, basal ganglia, and frontal lobes [1]. It can be caused by infections, immunosuppression, transplantation, connective tissue abnormalities, uremia, and hypertensive crises [2,8]. To capture both typical and atypical cases of PRES, Fugate et al. proposed the following steps for the diagnosis of PRES: one or more acute neurological symptoms described above, one or more risk factors such as severe hypertension or blood pressure fluctuations, renal failure, immunosuppressant therapy or chemotherapy, eclampsia or autoimmune disorder, and finally, brain imaging that could show bilateral vasogenic edema, cytotoxic edema with pattern of PRES or even be normal [9]. While the precise pathophysiological process behind PRES is unknown, one leading hypothesis postulates that quickly developing hypertension can cause the blood-brain barrier to break down through hyperfusion resulting from the cerebral blood flow autoregulation mechanism mounting an insufficient response [9].

Systemic lupus erythematosus (SLE) is a chronic autoimmune disease with clinical features ranging from mild skin rash to severe organ damage [10]. It is a multisystem disease and can affect the joints, brain, lungs, kidneys, and blood vessels of the patient [11]. SLE most commonly affects females and in particular females of childbearing age [10]. When treating SLE, hydroxychloroquine is the first drug of treatment with glucocorticoids being used to address flare-ups of the disease [10]. Higher doses of methylprednisolone are sometimes used in case there is a significant risk of organ damage [12]. Immunosuppressants are recommended if the patient doesn’t respond to the initial line of treatment or cannot take glucocorticoids within the recommended daily range for long-term use [13].

Antineutrophil cytoplasmic antibody-related vasculitis, psoriatic arthritis, systemic sclerosis, SLE with nephritis, and SLE without nephritis were among the rheumatic conditions linked with PRES (odds ratio (OR), 9.31, 4.61, 6.62, 7.53, and 2.38, respectively) [14].

The neurological system is affected by SLE in 12% to 95% of patients [15]. SLE-PRES patients frequently experience a significant rise in blood pressure, renal failure, and humoral retention, particularly when high doses of methylprednisolone or immunosuppressants are used to treat it. Some academics have thus hypothesized the interplay of the aforementioned elements to be the pathogenic mechanism of SLE-PRES. Autoimmune inflammation or ischemia alterations brought on by SLE (such as vasculitis, thrombosis, embolism, and vasospasm) could also result in PRES. Other researchers have suggested that rather than being immediately brought on by the underlying lesion of SLE, PRES should be seen as a subsequent consequence of SLE during treatment [16]. PRES is more common in lupus patients with poorly managed blood pressure, renal illness, or those on immunosuppressive medication [2].

In this systematic review, we aim to explore the relationship between hypertension and its possible role in the development of PRES in SLE patients.

## Review

Methodology

This systematic review was conducted using the Preferred Reporting Items for Systemic Review and Meta-Analyses (PRISMA) 2020 guidelines [17].

Search Sources and Strategy

We searched PubMed, PubMed Central (PMC), Multidisciplinary Digital Publishing Institute (MDPI), and Cochrane Library to search for the relevant literature. We used various combinations of SLE, PRES, and hypertension keywords to search all databases. We also used a MeSH strategy to query PubMed for relevant literature: (("Lupus Erythematosus, Systemic"[Mesh]) AND ("Hypertension"[Mesh])) AND ("Posterior Leukoencephalopathy Syndrome"[Mesh]). Table 1 shows the databases used and the identified numbers of papers for each database.

**Table 1:** Keywords/search strategy used and the number of identified papers MDPI: Multidisciplinary Digital Publishing Institute

Keywords/search strategy	Database used	Number of articles
(("Lupus Erythematosus, Systemic"[Mesh]) AND ("Hypertension"[Mesh])) AND ("Posterior Leukoencephalopathy Syndrome"[Mesh])	PubMed (MESH)	7
((hypertension) AND (posterior reversible encephalopathy syndrome)) AND (Systemic lupus erythematosus)	PubMed (Advanced Field search)	67
hypertension and posterior reversible encephalopathy syndrome and systemic lupus erythematosus patients	PubMed (Regular search)	52
hypertension and systemic lupus erythematosus	MDPI	23
hypertension and posterior reversible encephalopathy syndrome	MDPI	9
hypertension and posterior reversible encephalopathy syndrome and systemic lupus encephalopathy	Cochrane library	2
hypertension and posterior reversible encephalopathy syndrome	Cochrane library	37

Inclusion and Exclusion Criteria

Only papers written in English or those with a full-text English translation were included in our selection of articles. We only included research publications with human subjects.

In cases where the complete text of the papers could not be retrieved, articles were excluded. Gray literature as well as those that included pregnant people or age groups younger than 14 years were excluded.

Selection Process

The selected articles were relocated to Endnote (Clarivate Plc, Philadelphia, United States, London, United Kingdom), and any duplicate papers were eliminated. Each article was reviewed by looking at the titles and abstracts. Any disagreements about eligibility were discussed and resolved by general agreement. Only pertinent articles were reviewed when the shortlisted articles were given a full-text evaluation. Shortlisted articles were the only ones that met the inclusion and exclusion criteria.

Quality Assessment of the Studies

Using the appropriate quality assessment techniques, the papers that made the shortlist were evaluated for quality. The Newcastle-Ottawa method was used to rate the quality of observational studies, while the Assessment of Multiple Systematic Review (AMSTAR) tools were used to rate the quality of systematic reviews. For narrative reviews, the Scale for the Assessment of Narrative Review (SANRA) was used. The Joanna Briggs Institute (JBI) checklist was utilized to examine case reports. In this systematic review, only studies that met the quality appraisal criteria were considered.

Data Collection Process

After the articles were finalized for the systematic review and extracted, the primary outcomes were assessed along with other necessary information.

Results

Study Identification and Selection

**Figure 1:**
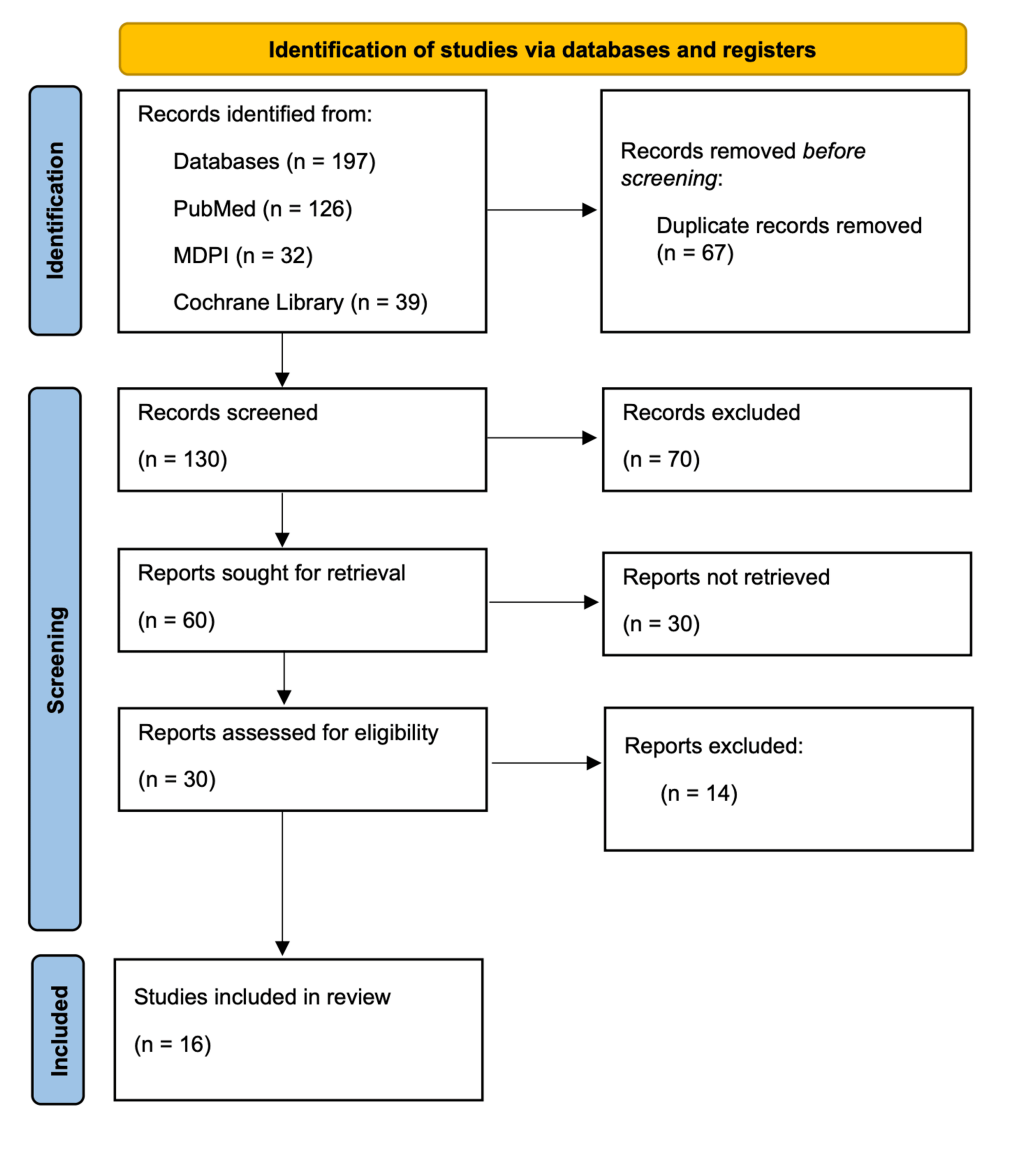
PRISMA flowchart showing the process of article selection. PRISMA: Preferred Reporting Items for Systemic Review and Meta-Analysis; MDPI: Multidisciplinary Digital Publishing Institute

We identified a total of 197 relevant articles using all databases. In total, 67 duplicate articles were removed before screening them in detail. After screening these articles by reviewing titles and abstracts and retrieving full texts, 30 articles were shortlisted. The shortlisted full-text articles were assessed for eligibility and quality, and 16 were finalized for review. The selection process of the studies is shown in Figure 1 in the PRISMA flowchart.

The articles were assessed for eligibility using the Newcastle Ottawa tool. Table 2 below shows the results of the quality appraisal.

**Table 2:** Quality appraisal using the Newcastle-Ottawa tool

Study	Selection	Comparability	Outcome
Chalico et al. 2018 [18]	****	*	**
Damrongpipatkul et al. 2018 [19]	****	*	***
Liu et al. 2012 [20]	***	*	**
Gatla et al. 2013 [21]	****	*	*
Hinchey et al. 1998 [1]	****	*	**

Case reports were finalized using the JBI quality check tool, and narrative reviews using the SANRA checklist.

Outcomes Measured

The primary outcome extracted from the finalized research papers was the association of hypertension with PRES in SLE patients. Other outcomes assessed were other risk factors of PRES and treatment of PRES in SLE patients. A few studies explored differential diagnoses of neuropsychiatric symptoms in SLE patients.

Study Characteristics

Table 3 includes a summary of the included observational studies.

**Table 3:** Summary of included observational studies PRES: Posterior reversible encephalopathy syndrome; SLE: Systemic lupus erythematosus; VEGF: Vascular endothelial growth factor; SLEDAI: Systemic Lupus Erythematosus Disease Activity Index

Author and year of publication	Number of participants	Purpose of the study	Results and conclusion
Chalico et al. 2018 [18]	32	This study aimed to determine whether patients with PRES-SLE have a specific blood cytokine profile. They also assessed soluble CD40L and VEGF, two markers directly linked to systemic endothelial injury.	PRES-SLE patients had a higher value of cytokine levels (IL-6 and IL-10) as compared to other patients. There was no correlation between cytokine levels with MRI abnormalities and PRES remission. PRES had no significant effect on other cytokine levels and CD40L and VEGF.
Liu et al. 2012 [20]	732	The current study's objectives were to identify the connection between PRES and SLE and look for an effective PRES treatment plan for SLE.	Ten occurrences of PRES in SLE patients were found. All of the patients were female, the mean age of onset was 22.93 2.48 years, and the SLEDAI was 25.8 5.7 at the time of the onset of PRES. Acute headache, changed mental status, stupor, vomiting, cortical blindness, and seizures were present in all instances. After timely corticosteroid treatment, full clinical and radiographic improvement was seen in eight patients. Along with more conventional causes like hypertension, lupus may cause PRES.
Gatla et al. 2013 [21]	5	To look for any distinctive clinical patterns in PRES patients since immune suppression would need to be increased rather than lowered or stopped in cases of neuropsychiatric lupus.	All exhibited hypertension, moderate to severe disease activity, and the standard MRI features of PRES and nephritis at the time of PRES presentation. The most frequent clinical symptoms were headaches, disorientation, and seizures.
Hinchey et al. 1998 [1]	15	It investigated through the log books for CT and MRI investigations conducted at the New England Medical Center in Boston, United States, and the Hôpital Sainte Anne in Paris, France, to better understand PRES. The study reported 15 patients who had been examined between 1988 and 1994.	Patients with renal insufficiency, hypertension, or immunosuppression may experience reversible, primarily posterior leukoencephalopathy. Neuroimaging results show subcortical edema without infarction, which is typical.

Table 4 contains a summary of the included case-cohort studies.

**Table 4:** Summary of included case-cohort studies PRES: Posterior reversible encephalopathy syndrome; SLE: Systemic lupus erythematosus; UPCR: Urine protein creatinine ratio

Author and year of publication	Number of participants	Purpose of the study	Results and conclusion
Damrongpipatkul et al. 2018 [19]	1332	This study aimed to identify the prevalence, clinical characteristics, brain imaging results, outcomes, and related factors of PRES in Thai SLE patients.	Anemia (recent hemoglobin 10 gm/dL) and high-degree proteinuria (UPCR > 1.0), which indicated underlying active nephritis, were discovered to be independent related factors of PRES. It is necessary to confirm whether anemia is a contributing factor to PRES.

Table 5 contains a summary of the included meta-analysis studies.

**Table 5:** Summary of included meta-analysis studies PRES: Posterior reversible encephalopathy syndrome; SLE: Systemic lupus erythematosus

Author and year of publication	Number of participants	Purpose of the study	Results and conclusion
Shaharir et al. 2013 [22]	87	This study aimed to describe the characteristics of SLE patients with PRES and the contributing variables to their poor prognosis.	Asians (74.2%) and women (95.4%) comprised most cases, with a mean age of 26.3 +- 8.8 years. Hypertension (91.7%), renal involvement (85.1%), and active illness (97.5%) were all significantly correlated with PRES. Involvement of the brainstem and intracranial bleeding were the two key indicators of a poor PRES outcome.

Table 6 contains a summary of the included narrative reviews.

**Table 6:** Summary of included narrative reviews PRES: Posterior reversible encephalopathy syndrome; SLE: Systemic lupus erythematosus; CNS: Central nervous system; RPLS: Reversible posterior leukoencephalopathy syndrome

Author and year of publication	Number of participants	Purpose of the study	Results and conclusion
Karoui et al. 2008 [23]	31	Compared the clinical characteristics and neuroimaging results of SLE patients with PRES.	Out of 31 patients, 27 had high blood pressure, 26 had lupus nephritis, 30 had seizures, and 23 had headaches, according to a comparison of clinical features.
Mak et al. 2008 [24]	17	The article aimed to quickly identify and distinguish RPLS from neuropsychiatric SLE (NPSLE) and lupus-related problems so that future therapeutic approaches and outcomes may be examined.	In contrast to the CNS alterations caused by lupus, RPLS requires reduced immunosuppressants, quick blood pressure, and seizure control to achieve complete neurological recovery. The advantages of immunosuppressant augmentation occasionally outweigh the hazards of having RPLS when lupus activity is high. Immunosuppressant dosages should be increased in these situations, and RPLS must be prevented from developing by carefully monitoring blood pressure, neuropsychiatric state, eyesight, and renal function.

A few case reports on PRES-SLE were also reviewed to find their association with hypertension. Table 7 displays the summary of the included case reports.

**Table 7:** Summary of included case reports BP: Blood pressure; ICP: Intracranial pressure; SLE: Systemic lupus erythematosus; PRES: Posterior reversible encephalopathy syndrome; RFT: Renal function test

Author and year of publication	BP (mmHg)	Summary
Sudan et al. 2022 [25]	220/120	A 32-year-old SLE patient presented with acute vision loss and other findings diagnostic with PRES. Her BP at the presentation was 220/120mmHg. She was treated with antihypertensives and switched from injectables to oral in the subsequent days, with complete resolution of her symptoms.
Hao et al. 2021 [16].	107/79 (normotensive)	The SLE patient, a 28-year-old female, who had a normotensive blood pressure of 107/79 mm Hg, had several episodes of seizures and was diagnosed with PRES. Treatments for dehydration, anti-epileptic drugs, and BP-lowering drugs were given.
Hartman et al. 2020 [26]	200/108	According to Hartman et al., PRES symptoms were identified in a 21-year-old female SLE patient with a confirmed BP of 200/108 mmHg. She recently stopped using her hypertension medication, and to treat her seizures and control her BP, she was also given IV lorazepam and labetalol.
Gauiran et al. 2018 [2]	190/110	A woman with previously diagnosed SLE who took her antihypertensive medication religiously and developed PRES symptoms with elevated BP of 190/110mmHg was documented. Following urgent medical ICP lowering medication and stringent BP control, the patient reported no return of the severe headache or convulsions at the six-week checkup.
Mani et al. 2016 [27]	160/98	Another SLE-PRES patient with a BP of 160/98mmHg in the Mani et al. review showed complete recovery from quadriparesis after initiating hemodialysis therapy, anti-edema medications, and antihypertensive medications along with cyclophosphamide and methylprednisolone in 10 days.
Sulaiman et al. 2011 [28]	160/110	A 33-year-old lady with undiagnosed SLE and other comorbidities came with acute glomerulonephritis, hypertensive emergency, and confusion. Cranial MRI revealed characteristics that are typical with PRES. At the presentation, the patient's BP was 160/110 mmHg. She eventually had her BP under control, with systolic readings between 120 and 140 and diastolic readings between 60 and 90 mmHg.
Morelle et al. 2009 [29]	155/100	Another patient, a 19-year-old woman with severe grade 4 lupus nephritis and SLE disease activity index of 39, was treated with hemodialysis because of deranged RFT but eventually presented with clinical features of PRES two weeks later at a BP of 155/100 mg and was managed on the lines of the PRES treatment protocol.
Patrick et al. 2003 [30]	170/100	Another study showed a patient in status epilepticus with cortical blindness at 170/100 mm Hg, suggesting PRES. She fully recovered once she started taking BP medicine. Three weeks later, she experienced another attack of seizures and cortical blindness as her BP rose to 150/90. After eight weeks, all neurological symptoms and signs vanished, renal function recovered, and mycophenolate mofetil was employed to manage BP and suppress the immune system aggressively. She still uses the immunosuppressant mycophenolate mofetil and manages her blood pressure perfectly.

Discussion

Pathophysiology, Causes, Clinical Features, and Investigation Findings of PRES

The precise pathophysiological process behind PRES is still unknown. Currently, three hypotheses have been put forth, including (i) cerebral vasoconstriction with subsequent brain infarcts, (ii) cerebral autoregulation failure with ensuing vasogenic edema, and (iii) endothelial damage with disruption of the blood-brain barrier causing fluid and protein transudation in the brain [20]. Various experimental experiments, neuroimaging, and post-mortem analyses support the latter two ideas. Byrom's experiment from the 1950s showed that a sudden rise in arterial blood pressure in rats led to functional vascular alterations that temporarily enlarged the posterior region of their brains. After the blood pressure returned to normal, the edema completely disappeared. The brain's vasculature automatically regulates to maintain a constant cerebral perfusion pressure (CPP) in response to abrupt increases in mean arterial pressure (MAP). This autoregulation is mostly accomplished through sympathetic nervous system-mediated compensatory cerebral vasoconstriction. A sudden rise in MAP that exceeds the autoregulatory ability of the cerebral vasculature might cause dilatation of the arterioles because the vertebrobasilar vasculature has somewhat less sympathetic innervation than the internal carotid artery system. Following arteriolar dilation, plasma, cells, and extravasate protein led to posterior cerebral edema and possibly additive endothelial harm from uremia and cytotoxic drugs [24].

**Figure 2:**
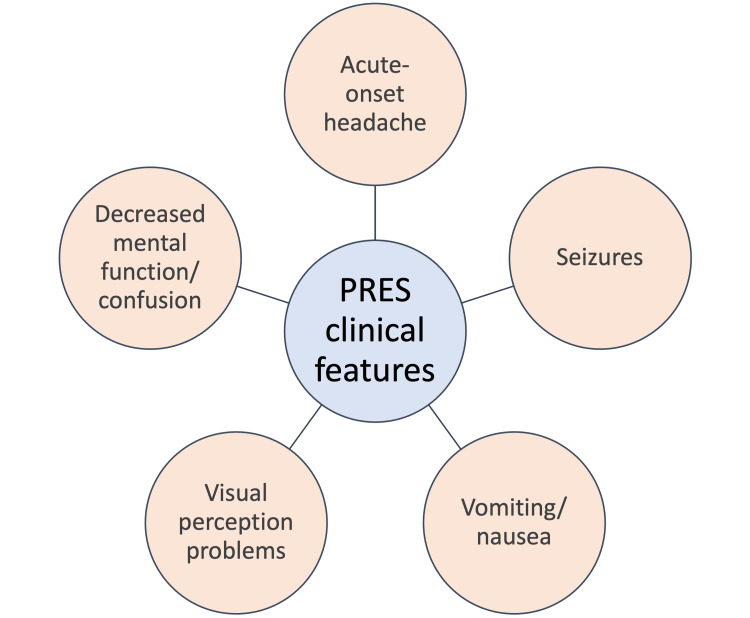
PRES clinical features PRES: Posterior reversible encephalopathy syndrome Figure Credit: Author Japneet K. Bhangu

Acute-onset headache, vomiting, seizures, abnormalities in visual perception, and alterations in the parieto-occipital white matter on MRI are all signs of PRES, which is both a clinical and radiological entity [2]. PRES patients can also show signs of quadriparesis with spasticity in limbs and hypertonia in all the limbs with extensor plantar response [27]. Figure 2 below highlights the typical clinical features of PRES.

Apart from the causes mentioned already, a few cases of PRES have been linked to procedures like angiography and cardiac catheterization with IV contrast, the implantation of a left ventricular assist device (LVAD), neurosurgery, and measles vaccination [24]. Immunosuppressants like cyclosporine and tacrolimus can cause PRES through several routes without significantly raising blood pressure. In addition to calcineurin inhibitors, cisplatin, IV Immunoglobulins, cytarabine, L-asparaginase for treating acute lymphoblastic leukemia, and bevacizumab monoclonal antibody for treating colon cancer were also described as frequently reported drugs that cause PRES [24].

WBC counts greater than 9 x 106, urine protein to creatinine ratio greater than 1, hemoglobin lower than 10 g/dL, cerebral hemorrhage, and brainstem involvement are risk factors for worse outcomes in PRES patients [26].

The first line of diagnosis for this illness is a head MRI [16]. Repeated and advanced neuroimaging may be considered if standard MRI results were normal or failed to explain neuropsychiatric signs and symptoms. As a result, for PRES patients, the appropriate scan should be done at the appropriate time [20]. PRES could develop even in the absence of severe hypertension due to the cytotoxic effect of SLE. Consequently, PRES may show as the first sign of SLE rather than a side effect of treatment [20].

Association of Hypertension With PRES in SLE Patients

In most instances, PRES is connected to hypertensive emergencies though this isn't always the case. The etiology of PRES has been connected to hypertension, which increases cerebral blood flow and eventually breaches the blood-brain barrier, creating vasogenic edema in the cortex [25]. High BP isn't always recorded in PRES, though. Even when drug levels are within the therapeutic range, immunosuppressive or cytotoxic drugs can have a direct toxic effect that can cause endothelial damage, decreased tissue perfusion, cytotoxic edema, blood-brain barrier disruption, and vasogenic edema. It's interesting to note that vasogenic edema can turn cytotoxic and cause cerebral infarction [23]. While the precise pathological mechanism for PRES in SLE patients is not clear, the aforementioned process involving hypertension in combination with endothelial damage and autoimmune activation, which SLE patients are at higher risk for, could potentially explain part of the pathophysiology [26].

We compiled and analyzed different patients with PRES-SLE and the association of elevated blood pressure among them. Table 8 summarizes the findings of a few studies that observed hypertension in SLE patients presenting with PRES.

**Table 8:** Studies evaluating the presence of associated factors and elevated BP in SLE patients presenting with PRES LN: Lupus nephritis; BP: Blood pressure; SLE: Systemic lupus erythematosus; PRES: Posterior reversible encephalopathy syndrome

Author name	Number of patients or episodes (N)	Elevated BP	Associated factors
Karoui et al. [23]	31	87%	LN 96%
Gatla et al. [21]	5	100%	LN 80%
Shaharir et al. [22]	87	91.7%	LN 85.1%
Damrongpipatkul et al. [19]	30	96%	LN 86%
Liu et al. [20]	10	80%	Renal failure (Cr>1.5) 20%

Reviewing the literature above, we observed that most of the previously described patients of SLE with PRES had severe hypertension (>170/110 mmHg) and renal failure.

We discovered the following findings regarding their BP as indicated in Table 9 below after analyzing the data from two studies that distinguished PRES-lupus patients from PRES caused by other reasons.

**Table 9:** Comparison of blood pressure readings in lupus patients presenting with PRES and those that presented with PRES but did not have lupus. PRES: Posterior reversible encephalopathy syndrome

Author name	Condition	Number of patients (N)	Number of patients with high blood pressure	Mean systolic blood pressure (mmHg)	Mean diastolic blood pressure (mmHg)
Mak et al. [24]	PRES with lupus	17	16	187.6	113.5
Hinchey et al. [1]	PRES without lupus	13	10	159.4	93.6

Another similar study compared the blood pressure readings between patients with PRES-lupus and patients with lupus only. They observed elevated blood pressure (>150/90 mmHg) in five out of 14 PRES-lupus patients compared to only one patient having hypertension out of six lupus patients [18]. In the above three studies [24,1,18], we observed a link between PRES and hypertension and increased hypertension severity in PRES-lupus patients compared to PRES patients without lupus. But at the same time, the severity of hypertension is not significantly associated with the intensity of the clinical and radiological manifestation of PRES. 

Similarly, on analyzing the case reports literature of eight patients of SLE presenting with PRES, we found seven patients to have elevated blood pressure (>150/90mmHg) and only one patient to be normotensive. All patients were further treated with antihypertensive medications and other supportive treatments [16,25-30]. In one report, the patient had a recurrence of symptoms after full resolution once when her blood pressure rose again, and she was treated again to normalize her blood pressure [29]. Hence, blood pressure maintenance is important during the course of the disease for SLE patients and should not be ignored.

Role of PRES in SLE Patients

In a case-control study, the prevalence of PRES was shown to be up to 0.43% in patients with SLE. Although PRES is uncommon among SLE patients, it is linked to a high mortality rate [26]. Therefore, it is important to understand PRES in SLE patients and its association with hypertension.

PRES has been noted in lupus patients, particularly those with immunosuppressive medication, renal illness, or poorly managed blood pressure [2]. When high doses of methylprednisolone or immunosuppressants are used to treat serious diseases, patients with SLE-PRES frequently display a significant rise in blood pressure, renal failure, and humoral retention [16]. A study of 98 patients with SLE and PRES was recently analyzed in three retrospective reviews [29]. Although the beginning of PRES was associated with an SLE flare-up in more than 90% of cases, other variables such as hypertension (82-95%), renal insufficiency (73-84%), and the use of immunosuppressive medications (50%) were frequently present [29].

In patients who had PRES, the SLE Disease Activity Index (SLEDAI) criteria for lupus was greater (by about six points), indicating a more severe case of the disease at the time of diagnosis [26]. Additionally, renal impairment, hypoalbuminemia, and thrombocytopenia are independent risk factors for PRES and may be related to SLE [26].

In a study, J. Merayo-Chalico et al. examined the expression of various serum cytokines such as IL, as well as vascular endothelial growth factor (VEGF) and soluble CD40 ligand (sCD40L), in PRES-SLE patients and contrasted those levels with levels in SLE patients without PRES and in healthy controls. They analyzed the reports of 32 people (14 PRES-SLE patients, six healthy controls, six SLE patients in remission, and six SLE patients with active disease). They discovered that PRES-SLE patients had significantly greater IL-6 and IL-10 levels than the other groups (P = 0.013 and 0.025, respectively). Additionally, there was a positive association between the levels of IL-6 and IL-10 (r = 0686, P = 007). Regarding the levels of sCD40L, VEGF, or other cytokines, there were no variations between groups [18].

Treatment of PRES in SLE Patients

PRES might not always be completely reversible, despite the name. According to data available on the consequences of PRES, there have been cases of cerebral infarction, subarachnoid hemorrhage, coma, and death. Most of PRES's management is supportive. Another crucial PRES management component is a treatment that addresses the underlying cause [2].

Patients with lupus-related PRES should have a 10-25% reduction in MAP or a diastolic blood pressure reading of less than 100 mmHg within the first two hours [24]. A target mean arterial blood pressure between 105 and 125 mmHg has been suggested [23]. Parenteral antihypertensive drugs should be used to quickly lower blood pressure while closely monitoring it to avoid hypoperfusion, as a blood pressure drop that occurs too quickly can lead to hypoperfusion and cause end-organ damage such as cerebral infarction, abrupt myocardial infarction, and renal shutdown [23,24]. Using nimodipine, a calcium channel blocker, is potentially helpful in preventing cerebral vasospasm [23]. The selection of antihypertensive medications for SLE patients with lupus-related PRES should be cautious because several current antihypertensive medications, such as hydralazine and methyldopa, might cause drug-induced lupus and are therefore inappropriate for SLE patients. In ICUs where close hemodynamic monitoring is easily accessible, IV antihypertensive medications like nitroprusside and labetalol (with alpha and beta blockade activity) are favored [24].

Anti-epileptic drugs should be used to manage acute seizures, no matter their cause. This should be done until the PRES symptoms stop manifesting [2]. Phenytoin or carbamazepine should not be used to treat PRES-related seizures in SLE patients because they can lead to drug-induced lupus and complicate the clinical picture of the patient's pre-existing lupus [24].

PRES can be brought on by using corticosteroids and immunosuppressive drugs, according to the findings published by Mak et al., who also found that these medications' administration needs to be discontinued immediately when this happens. Although PRES may indicate lupus activity, IV methylprednisolone, and cyclophosphamide are still the most often prescribed medications for people with lupus activity [24].

If someone has a significant fluid retention problem, hemodialysis may be necessary [2]. Another SLE PRES patient with a blood pressure of 160/98 in the Mani et al. review showed complete recovery from quadriparesis after initiating hemodialysis therapy, anti-edema medications, and antihypertensive medications along with cyclophosphamide and methylprednisolone in 10 days [27]. Her PRES was triggered due to grade 4 lupus nephritis and not high blood pressure, which led to fluid retention. 

In a similar study, another patient, a 19-year-old woman with severe grade 4 lupus nephritis and SLE disease activity index of 39, was treated with hemodialysis because of deranged RFT but eventually presented with clinical features of PRES two weeks later [29]. This shows that along with elevated BP, other factors like deranged kidney function, abnormal kidney biopsy, and SLEDAI severity also play an important role in PRES development. They should be kept in mind while evaluating and treating patients. Furthermore, intracranial bleeding (OR 14, 1.1-187.2, P = 0.04) and brainstem involvement (OR 10.9, 1.3-90.6, P = 0.003) were found to be predictive of a poor outcome in PRES patients [22].

In patients with PRES whose seizures and hypertension are poorly controlled, irreversible lesions can develop due to the transition from vasogenic to cytotoxic edema, indicating a change into intracerebral hemorrhages and infarcts, ultimately resulting in lifelong neurological impairment [24]. At the same time, despite a quick drop in blood pressure, a patient with SLE PRES in another evaluation did not entirely recover vision. Hence the word reversible may consequently be misleading because 50% of cases may result in persistent deficiency, particularly in the area of vision [28].

Differential Diagnosis of PRES in SLE Patients

Imaging results and reversibility are major factors that help in separating PRES from other possible diagnoses, such as bilateral ischemic strokes in the posterior cerebral artery territory, central venous sinus thrombosis, demyelinating diseases, lupus encephalitis, cerebral vasculitis, and infectious or metabolic encephalopathy, which are all common in SLE patients, and prevent unnecessary extra testing [2,23]. The primary differential diagnosis of PRES is bilateral ischemic strokes in the posterior cerebral artery region. This distinction is significant because, while blood pressure should not be aggressively addressed in cases of cerebrovascular infarction, care of PRES requires quick control of blood pressure [23].

In lupus patients, PRES, neuropsychiatric SLE (NPSLE), and CNS problems can occur, and their clinical picture overlaps in most cases; hence, it is frequently difficult to distinguish between these conditions, especially in the early stages. The likelihood of PRES significantly increases in lupus patients with PRES-like neurological symptoms when the characteristic symptoms of PRES are promptly recognized, with special attention paid to the recent start or augmentation of immunosuppressive medicines. Thus, in addition to urgent neuroimaging, a thorough physical examination, checking for the focal neurological deficit, and a mental state examination are always the cornerstones in diagnosing PRES. The history should be carefully taken, with questions about headaches that recently started, seizures, visual disturbance, and recent changes in medication. After carefully ruling out other illnesses, an MRI of the brain with PRES-specific abnormalities leads to the correct diagnosis of PRES. Immunosuppressive therapy should be started or increased along with the appropriate auxiliary treatment such as antiepileptics or anti-psychotics for lupus patients who present with neuropsychiatric symptoms without particularly abnormal focal neurological signs, blood screening, cerebrospinal fluid (CSF) analysis, or neuroimaging findings because of the likely diagnosis of NPSLE [24].

The likelihood of corticosteroid-induced psychosis should also be considered in patients with active lupus who have just started taking or increased their corticosteroids, especially if the corticosteroid dose is high. Reducing the dosage of corticosteroids while closely monitoring neuropsychiatric symptoms and lupus activity is frequently effective in differentiating between NPSLE and corticosteroid-induced psychosis [24]. 

To rule out further central nervous system (CNS) disorders such as infection, demyelination, cerebral vasculitis, and subarachnoid hemorrhage, lumbar puncture and CSF analysis can be used [24]. Thrombotic thrombocytopenic purpura (TTP) should also be considered in lupus patients who report an altered neurological state, fever, microangiopathic hemolytic anemia, and renal impairment and should be further treated with plasmapheresis [24].

One of the pathological characteristics of NPSLE is the activation of endothelial cells. It typically happens after exposure to IL-1 and TNF-alpha; local release of IL-1 and IL-6 may worsen it. The blood-brain barrier (BBB) is damaged, and plasma leakage occurs in SLE patients with high SLEDAI due to elevated serum levels of TNF-alpha and other pro-inflammatory cytokines that may activate astrocytes and intracranial artery endothelial cells to create nitric oxide (NO) [20].

Limitations

There is very little information about PRES, and even less information is accessible on SLE patients diagnosed with PRES. Even after a thorough search, no randomized clinical trials could be retrieved. Heterogeneity between studies is another limitation given we were doing a systematic review and the studies included had different study designs, number of participants, etc. It is also important to acknowledge the limitation imposed by relatively small sample sizes of patients in the included studies since that limits the statistical power. Regarding the connection between PRES-SLE and hypertension, although hypertension was found to be the predominant association in PRES-SLE patients, other parameters, such as the severity of SLE illness, immunosuppressant use, and lupus nephritis, were also found to be associated with it. Therefore, a study that compared all the relevant variables and reduced them to one primary explanation was deficient, which could have influenced the findings. Larger prospective studies are required to define the etiology of PRES in SLE patients' treatment options and the risk of a bad outcome among them.

## Conclusions

An unrecognized neuropsychiatric manifestation in SLE patients is PRES. It is difficult to diagnose and treat PRES since it might be a symptom of active lupus illness or a side effect of immunosuppressant therapy, obscuring the particular role of SLE itself in developing PRES. However, we discovered a common factor among all of the patients we were able to recover from the data we reviewed: hypertension. All patients who presented with PRES had elevated blood pressure findings, albeit it was unclear if this was their first episode of elevated blood pressure or if they were previously taking antihypertensive medication and had adhered to it. However, certain SLE individuals may present with conditions that mirror how PRES presents; as a result, it's critical for clinicians to make the correct diagnosis by understanding the clinical aspects and neuroimaging findings of PRES for quick recognition of the condition and subsequent therapy. Aggressive treatment with antihypertensives and other medications should be initiated as soon as PRES is diagnosed. Clinicians should always keep PRES as a differential in neuropsychiatric lupus patients and be aware of its associations since prompt detection and treatment of PRES are crucial in preventing ischemia/infarction and long-term neurological impairments. For future research, it would be good to have clinical studies to further refine the best treatment option for SLE patients presenting with PRES.
