# Correction: The Association of Hypertension With Posterior Reversible Encephalopathy Syndrome in Systemic Lupus Erythematosus Patients: A Systematic Review

**DOI:** 10.7759/cureus.c254

**Published:** 2025-08-24

**Authors:** Japneet K Bhangu, Khalid Javed, Prabhleen Kaur Manshahia, Shamsun Nahar, Srishti Kanda, Uzair Chatha, Victor A Odoma, Aakanksha Pitliya, Esraa M AlEdani, Safeera Khan

**Affiliations:** 1 Internal Medicine, California Institute of Behavioral Neurosciences & Psychology, Fairfield, USA; 2 Anesthesiology, California Institute of Behavioral Neurosciences & Psychology, Fairfield, USA; 3 Internal Medicine/Family Medicine, California Institute of Behavioral Neurosciences & Psychology, Fairfield, USA; 4 Medicine, All India Institute of Medical Sciences, Rishikesh, IND; 5 Internal Medicine, Jean-Charles Medical Institute, Orlando, USA; 6 Medicine, California Institute of Behavioral Neurosciences & Psychology, Fairfield, USA; 7 Research, California Institute of Behavioral Neurosciences & Psychology, Fairfield, USA; 8 Cardiovascular/Oncology (Acuity Adaptable Unit), Indiana University Health, Bloomington, USA; 9 Dermatology, California Institute of Behavioral Neurosciences & Psychology, Fairfield, USA

This article has been corrected to fix the following typo in the PRISMA diagram: "Reports not retrieved (n = **39**)" has been corrected to "Reports not retrieved (n = **30**)".

